# Integrating the traditional Emirati community within urban fabric of the city to enhance the historical identity

**DOI:** 10.1016/j.heliyon.2021.e08650

**Published:** 2021-12-22

**Authors:** Inshirah Shublaq, Abeer Abu Raed, Tamarah Alqalami, Esraa Altwassi, Rafael Pizarro

**Affiliations:** a Department of Architecture, American University of Ras Al Khaimah, Ras Al Khaimah 10021, United Arab Emirates; b Department of Architectural Engineering and Technology, TU Delft, the Netherlands; c Department of Reconstruction and Projects, University of Baghdad, Iraq; d Istanbul Okan University, Faculty of Art, Design and Architecture, Department of Architecture, Turkey; e College of Architecture, Art and Design, Department of Architecture, American University of Sharjah, United Arab Emirates

**Keywords:** Heritage, Green fingers, Urban development, UAE

## Abstract

The site of Al Ain Civic Centre consists of major vacant lands and scattered development. This site is facing many issues such as car dependency, disconnectivity, low-density development, poor quality of life, and lost identity. Even though Al Ain city is well known for its green and open spaces, yet still such spaces cannot serve this site efficiently. This is because of the poor connectivity among open spaces and oases in Al Ain. One of the suggestions is to provide major connectivity plans and functional open spaces to solve such a situation and revitalize the area as a requirement made by Abu Dhabi Urban Planning Council. This is achieved through the demonstration of green fingers in a way that collects/integrates the site as a whole with the surroundings. As a result, introducing green fingers is part of planned solutions as well as a requirement by the Abu Dhabi Urban Planning Council. Another issue is the major breakage that is represented by Al Slimi wadi. This wadi is known for its transverse site as well as its disconnected pedestrian walkways. Hence, connecting the two areas by a pedestrian bridge is one of the solutions presented in this research. More importantly, the site is considered to handle a neighborhood development with a challenge that aims to preserve, maintain, and protect the traditional Emirati housing. This kind of housing was demonstrated in Fareej form with all its required elements such as courtyard houses, Sikkas, barahas, and maidan. The project turned out to represent a master-planned community. The site claims to work as a zero-car neighborhood, with allocated accesses that lead to a parking space and a golf cart rental station. In conclusion, this neighborhood site plan/the design of this neighborhood can provide an efficiently designed urban plan that is highly walkable, friendly, accessible, and simultaneously protects the environment for its users. The concepts of connectivity and proximity are applied successfully while maintaining the heritage side and Identity of Al Ain.

## 1 Introduction

**Figure 1:**
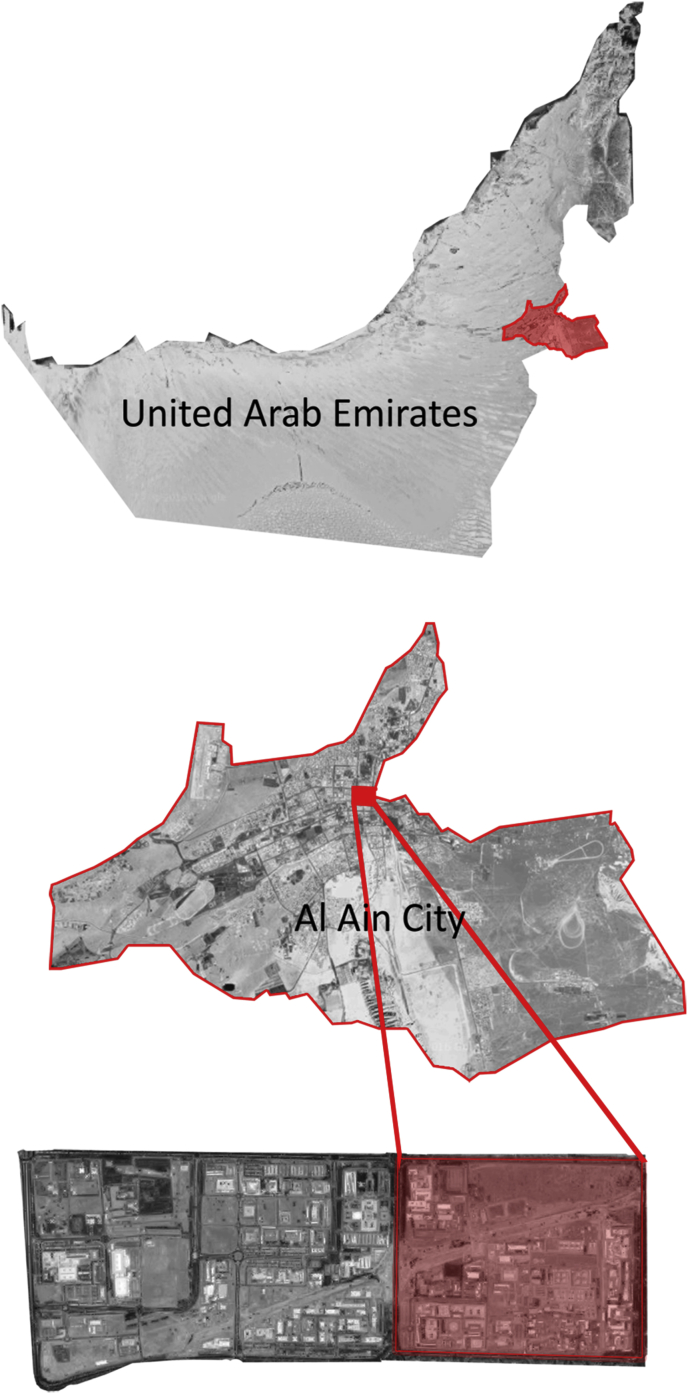
Site's location.

**Figure 2:**
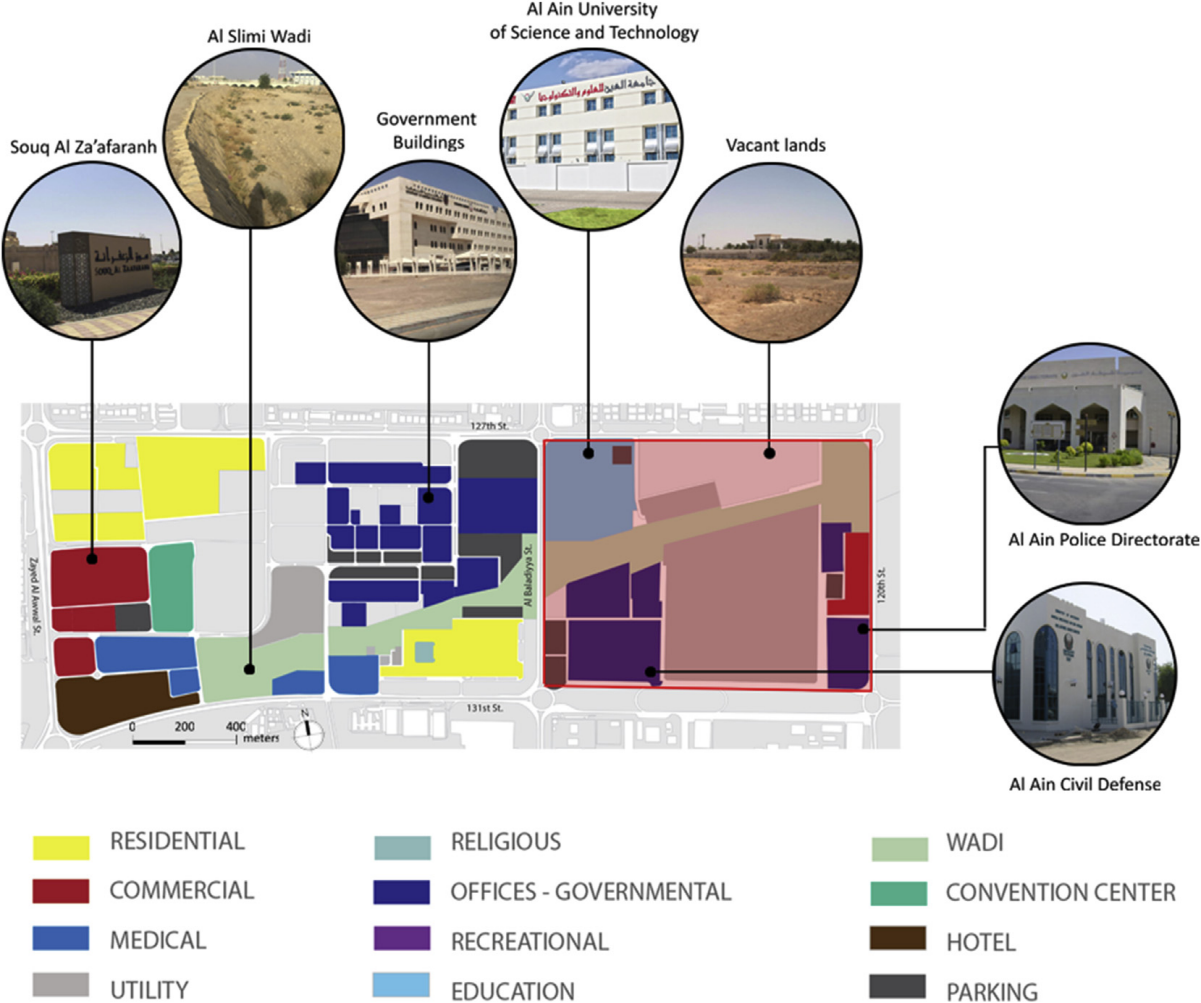
Existing land use. Almost half of the Civic Centre goes to vacant land and utility areas. Almost 20% of the area is dominated by the presence of the Wadi. The rest of the area gathers different uses (residential, commercial, medical, governmental, and recreational).

Due to the cultural uniqueness of Al Ain, it was inducted into the UNESCO World Heritage List. The inclusion was due to unique cultural sites, the historical importance of Jebel Hafit, Hili cultural landscape, Bidaa Bint Saud, the Oases areas and the Falaj system. UNESCO World Heritage Committee, held in June 2011 over its 35th session, registered this property for the World Heritage List. This research adopts a new approach that identifies cultural and historic entities as urban landscapes. The research approach emphasizes the importance of an inter-twined design of spatial planning and strategic planning. For urban areas with a history, the same approach has been suggested by the United Nations Educational, Scientific and Cultural Organization (UNESCO) and International Council on Monuments and Sites (ICOMOS) [1]. According to the Washington Charter [2], economic, cultural, and social development policies are integrated into projects of urban heritage preservation and regeneration. Thus, this research acknowledges the need for a link between heritage management and spatial planning. The goal of this work is to enhance the historical identity of Al Ain through revitalizing the traditional Emirati Fareej and neighborhood while connecting the city through greenways called (green fingers). The area is transverse by a major natural feature represented by Al Slimi Wadias as shown in Figures 1 and 2. The land indicated by red is for the exact site used for designing the project. Any adjacent area is part of the context of Civic Centre sharing the same urban fabric, qualities and identity but not part of the proposed area in the final master plan model. The selection was based on the percentage of built up area with least disturbance. The allocated red site was almost vacant.

Hence, these components should not be neglected, on the contrary, they all should be adapted to contemporary needs within a harmonious blend of relationships between traditional and contemporary cities to maintain a sustainable urban form.

Al Ain is a city that is affected widely by bio-physical conditions. Its location and identity show clearly the variety of natural elements Al Ain accommodates. The city of Al Ain lies at the meeting point of three different topographies (Jabil Hafeet, the nurturing oases and the majestic desert). Its landscapes and ecosystems, particularly the Oasis, Mountains, and Wadis, made it very diverse in wildlife (The Eastern Region's biodiversity is concentrated in Jebel Hafeet with 95%). Al Ain city includes virgin inhabitants, large cultivated farms, small patches of garden and open spaces, Wadi, etc. However, the green spaces available in Al Ain are scattered and not serving the users well. The current average walking time to open space is 10 min. Connecting these open spaces to make them accessible for users is the key to revitalizing them and reduce the average walking time to open an open space to 3 min. Open spaces should be treated at the micro-level in relation to the environmental qualities that tend to be treated at the macro level.

The Heritage and conservation field is widely known for its integrated relationship with urban planning since the early 1990s. This is because the basics of design strategies have developed an appropriate approach that maintains an integrated relationship between the responsibility of preserving cultural heritage and the demands to achieve the sustainability of development [3]. More importantly, Any conservation effort in historic towns must take place within the planning process [4]. Equally relevant, conservation and revitalization should be applied in harmony and not in a way that contradicts the logic of heritage identity and culture. The European Charter of the Architectural Heritage introduced this approach as a new policy in 1975 and became accepted as a theme in the integrated conservation approach [5, 6].

This research aims to highlight certain elements within a flexible design strategy to be applied in the sustainability as well as the conservation of city identity. These are demonstrated in green fingers and Fareej neighborhood and elements. The concepts selected to discuss the suggested contemporary design are connectivity and proximity. The research adopts a sustainable framework to protect and respect the place identity through well-managed sites.

Al Slimi Wadi transverses the site under study and works as a main feature in the site area. This wadi runs through Al Ain and it is dried out most of the year. Similarly, an appropriate strategic approach is the one that realizes the importance of avoiding the exploitation of the heritage for the sake of urban regeneration [7]. Thus, an initiative of creating a green finger to run along the wadi can work towards a macro level of major green connection throughout Al Ain. All public open spaces, including cultural landscapes, agricultural landscapes, parks and natural landscapes should be treated as part of a coherent whole. 1.7% of Al Ain city is public open space. It has 234 ha of developed parks for a population of nearly 623,000 (Plan Al Ain 2030).

**Figure 3:**
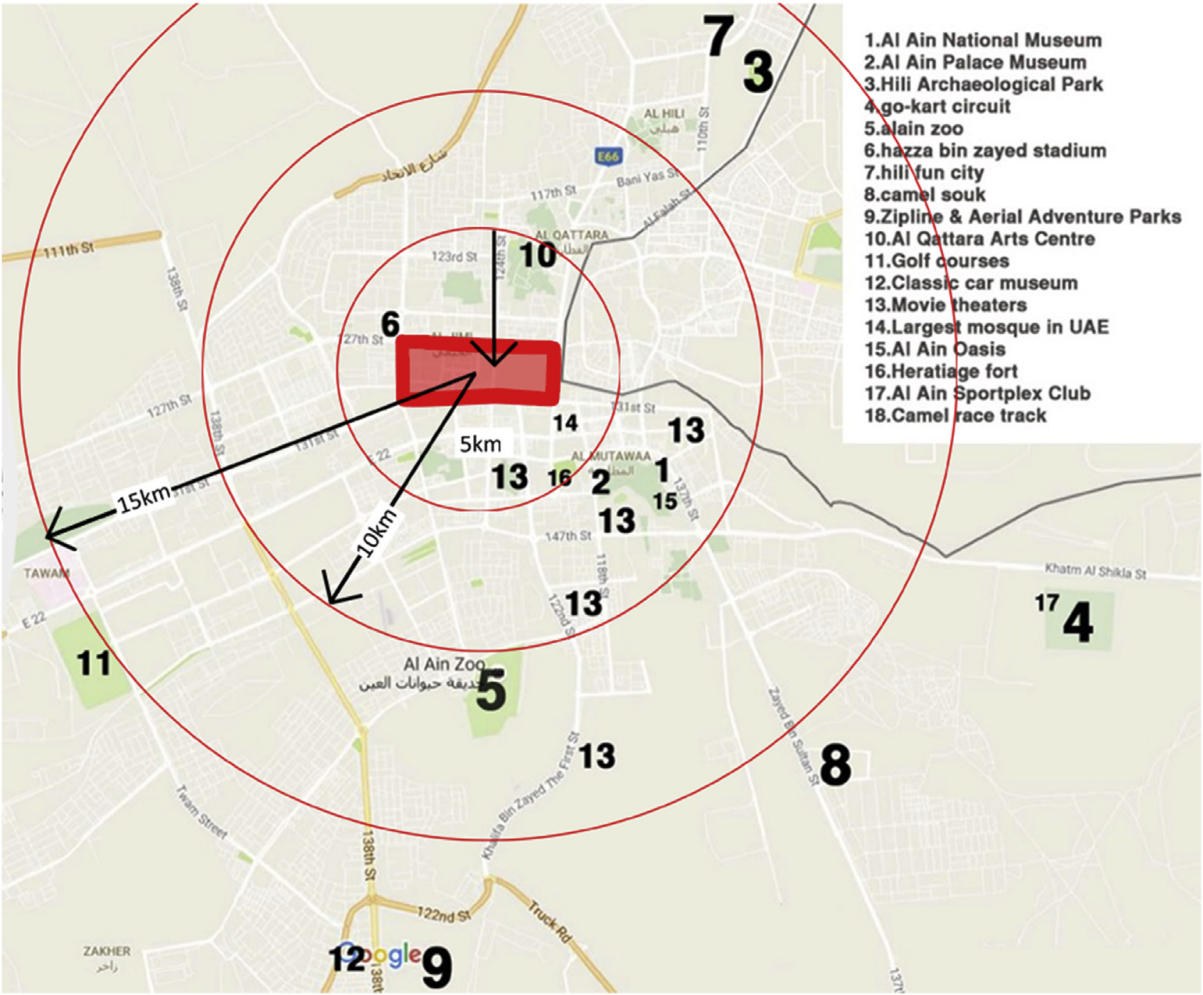
Attractions within the city.

Al Ain has numerous Attractions and activities as shown in Figure 3. Upon the conducted site visits and further site analysis, it was noticed that there is a huge gap between what the city offers the site under study and what families living in the site should be provided. The uniqueness of the site makes distributing the facilities and day-to-day activities challenging. Focusing on how to provide such amenities with proximal distances within the site will increase the chances of users to walk and enjoy their trips.

According to the regulations illustrated by UNESCO, strategic decisions are the reason behind the transformation of conservation decisions into regulations and establishing local standards [8]. It is argued that the future should always be part of the culture but not be imprisoned by the past/to exist only as an exact copy of the past [9].

In addition to the traditional causes of decay, historic urban fabric is also increasingly affected by changing social and economic conditions. In conclusion, A thorough study and development of the Plan of Al-Ain is still needed to address how tradition and modernity interact. The aim of this research is to develop an integrated relationship between the past and future by creating the context of a sustainable urban form based on a unified vision that combines tradition, modernity, and sustainability. The inconsistency of such relationships may result in many issues that affect the communication of the values, ideas, needs and identity of Al Ain community.

**Figure 4:**
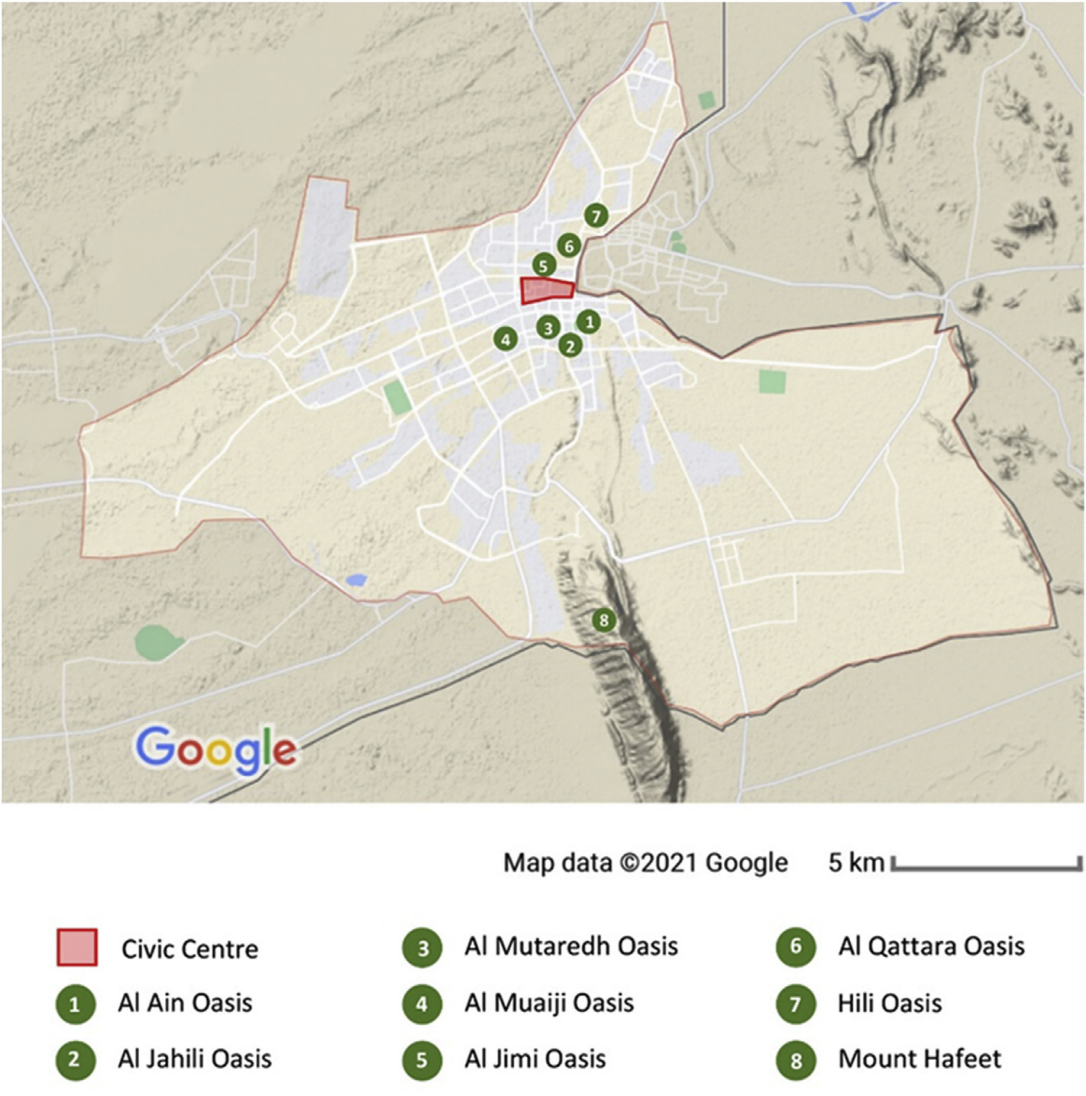
Al Ain Terrain showing the seven oases, the mountain and their relationship to the civic centre.

The UAE is a desert country and Al Ain is called “Garden city of The Gulf”. As shown in Figure 4, the city has mainly seven oases, Al Ain, Al Jahili, Al Mutaredh, Al Muaiji, Al Jimi, Al Qattara, and Hili. Al Ain Oasis is the largest while Al Jahili Oasis is the smallest. These oases are lined with palm trees. The city is known for its greenery, natural springs, and moderate climate compared to the rest of the emirates. The tallest mountain in Abu Dhabi, Mount Hafeet is located in Al Ain.

The only natural feature occurring in the Civic Centre is the dried-out Wadi. Other main bio-physical varieties (oases and mountain) are not located within the site, they are rather located around the Civic Centre within 5 km diameter. All the seven oases are very close to the Civic Centre in a way that calls for a certain layer of connection among them. The status-quo of the Civic Centre is a dry desert with almost 50% vacant land. The absence of any green natural elements called for the proposal of Green Fingers. Green Fingers work as added green tissue to connect these scattered oases and enhance the green image of the city.

## 2 Literature review

There is a growing need for enhancing the identity of cities in the gulf region, while preserving their authentic and historical features [10]. It is believed that heritage preservation works as an ally for economic and social development [11]. The challenge of having a revitalized historical urban space that is well connected within the rest of the city can't be undermined. The connection of the city with the revitalized space should happen at multiple levels. Connecting the city to the newly revitalized urban space should depend mainly on the nature of the city. In case that a city has urban natural resources, most of the connections might happen at the level of landscape [12]. By this ideology, a conflict between creation and preserving is settled to impose a unique product for the users to live the experience of an authentic space within a developed city.

Regulations and laws are inevitable in revitalising the architectural heritage and urban plan. For instance, in the UAE many historical buildings have been demolished only for the need to create new business towers and shopping malls [13, 14]. Consequently, preservation is not just about the preservation of the buildings or landscapes, as much as preserving the place's cultural identity [15]. Protecting individual buildings was the traditional approach in the past; however, its focus has shifted to a more holistic approach that focusses on areas. According to the “International Council on Monuments and Sites” (ICOMOS), the goal of conservation is to cultivate a place such that it sustains its “cultural significance”.

At the beginning and particularly when oil fields were discovered in the Arabian Gulf and later oil-driven urbanization and associated with rapid urban growth in population and infrastructure, the local authorities did not pay attention to the most important aspect which is preserving the features of the local identity and cultural values [16]. While in Western cities, especially, during the 1970s a decrease in massive projects that concern urban transformation is noticed while urban regeneration started as an alternative for urban renewal. For example, the “Amsterdam Declaration” incorporated cultural heritage into a city's conservation strategy and demonstrated its positive impact [17]. This is because revitalizing urban plans and forms adapts to the issues intending to improve the living standards for locals without losing the identity of place. It is a team effort in which even stakeholders must understand their city planning policies and regulations.

This research illustrates Al Ain city as a complex area that requires the protection of not just the architectural heritage within historic urban landscapes but also the protection of planning activities, nature, and documentation. In most cases, heritage conservation is considered an opposing concept to urban planning. Nonetheless, in most Western European countries these two terms represent two sides of the same coin [18]. Nevertheless, the same laws should not be presented as a rigid framework and outline. On the contrary, they should be elaborated in a way that works not just for protecting and preserving the heritage sites and identity but also for organizing the process of intervention and development to maintain economic and social patterns.

On one hand, there is always a misconception when it comes to the idea of understanding the heritage preservation consistency, particularly, in its role as a barrier that hinders any attempt to develop a historic urban landscape [19, 20, 21]. On the other hand, many attempts demonstrate the concept of heritage preservation as adaptable to change in a way that simultaneously serves economic and social development. Similarly, any city with a cultural heritage site and landscapes always has an advantage in the global market. This is because design strategy of heritage conservation projects a unique identity that can be easily integrated when developing local communities to accommodate the change in people's demands while preserving the historic urban areas [22]. Even though cities and communities are changed inevitably and sometimes transformed to meet the demands of their community, yet the conservation of historic fabric, pattern and elements is fundamental for protecting the urban identity and cultural values [23, 24, 25].

Therefore, there is a need to look at these elements as a tool to preserve history, culture, and nature while reconnecting between past, present, and future [26]. In this sense, UN-Habitat mentioned it is essential to start by preserving the cultural heritage in present cities by sustaining tangible and intangible aspects for their impact in shaping the place, identity and culture [27]. Regenerating cultural heritage serves as a means of enhancing a city's uniqueness and uniqueness as a whole.

## 3 Materials and methods

Although the traditional features of Al Ain doesn't call for a specific landscape design, the proposal of Green Fingers aligns with the need of green spaces within the city. On the one hand, this suggestion fulfills the urgent need of providing accessible green spaces for the users. There are two main layers of greenery in the proposed model, green open spaces and green connecting spaces. The green spaces can work in hierarchy as follows (from small to large scale):

1. Private dwelling courtyard
2. Pocket park/Baraha (shared among a fareej)
3. Neighborhood park/Maidan
4. Natural Oases

One the other hand, the green spaces already existing in Al Ain are mainly the oases, they surround the Civic Centre and need to be connected through another form of landscape. To ensure that the connectivity occurs at different levels, there are two main green connecting spaces as follows:

1. Green Fingers
2. Green Belts

The Green Fingers occur as a connective tissue between one pocket park and another and they lead to the common space that occurs in the neighborhood called Maidan. A Green Belt is the linear park that surrounds a single fareej. Both levels of connection are needed to ensure that a continuous green fabric occurs in the city. The absence of any green forms of landscape within the Civic Centre calls for connecting the scattered green oases already existing around it to enhance the image the city that is known for its greenery.

### 3.1 Alternatives and comparisons

Historically, the availability of a courtyard was a necessity in every dwelling unit and this element was called Hawsh. At a next level, an open space that occurs within one Fareej is called Barah. A larger common area that occurs among multiples of Fareej is called Maidan. The walkways that occur at a Fareej called Sikka. The same kind of elements can be reflected with similar hierarchy as contemporary elements. Below is a comparison table emphasizing the different terminologies of similar elements.

**Table:** 

Traditional element		Contemporary element
Fareej	→	Community
Hawsh	→	Private courtyard
Baraha	→	Pocket park
Maidan	→	Neighbourhood park
Sikka	→	walkway

Ideally, a Green Finger is a planted walkway. Traditionally, walkways were called Sikkas and they were randomly lined with palm trees. Green Fingers are an added layer to the Sikkas and can work as a connective element. They can work side by side within the Fareej to add a layer of sustainable aspect to the project.

This work supports the major aims of “Plan Al Ain 2030” that takes into consideration the increasing need for housing that doesn't only have traditional aspects but also respects the environmental and social considerations, as well as accessibility to transport and amenities. The suggested model in this project takes into consideration the traditional style of neighborhood in the UAE.

**Figure 5:**
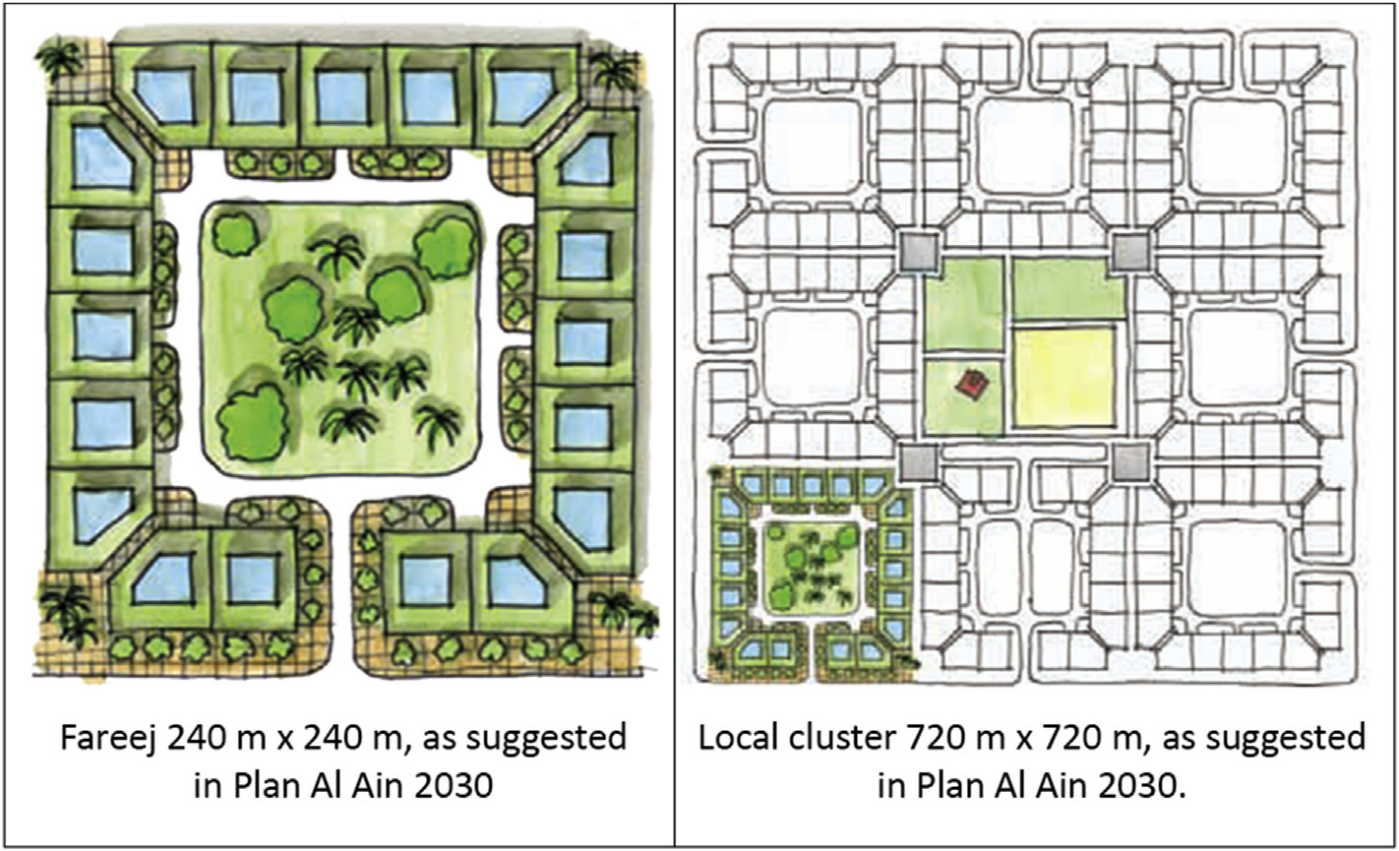
Abu Dhabi Urban Planning Council suggested design.

The smallest unit of the Emirati community is called “Fareej”. It is defined as a set of dwelling units that can be utilized to house an extended Emirati family, these units are normally clustered around a courtyard (Plan Al Ain 2030). The walkways are shaded and they are locally called “Sikka”. They connect courtyards together for easy movement and accessibility. The idea of “Green Fingers” was also incorporate into the design. Green fingers are a connective fabric of green walkways that connect the oases and run through the city of Al Ain. They are meant to connect green areas like parks, courtyards and public outdoor spaces to result in a continuous tissue of shaded and walkable paths running through the city. This's supposed to enhance the character of Al Ain as a green city (Plan Al Ain 2030). The main focus should be given to families. Providing for families and designing for them was the main aim throughout this project. Taking into consideration the cultural, traditional constraints, connecting those families to the surrounding world while providing for them public facilities within a walking distance. The suggested design by Abu Dhabi Urban Planning Council is presented in Figure 5.

**Figure 6:**
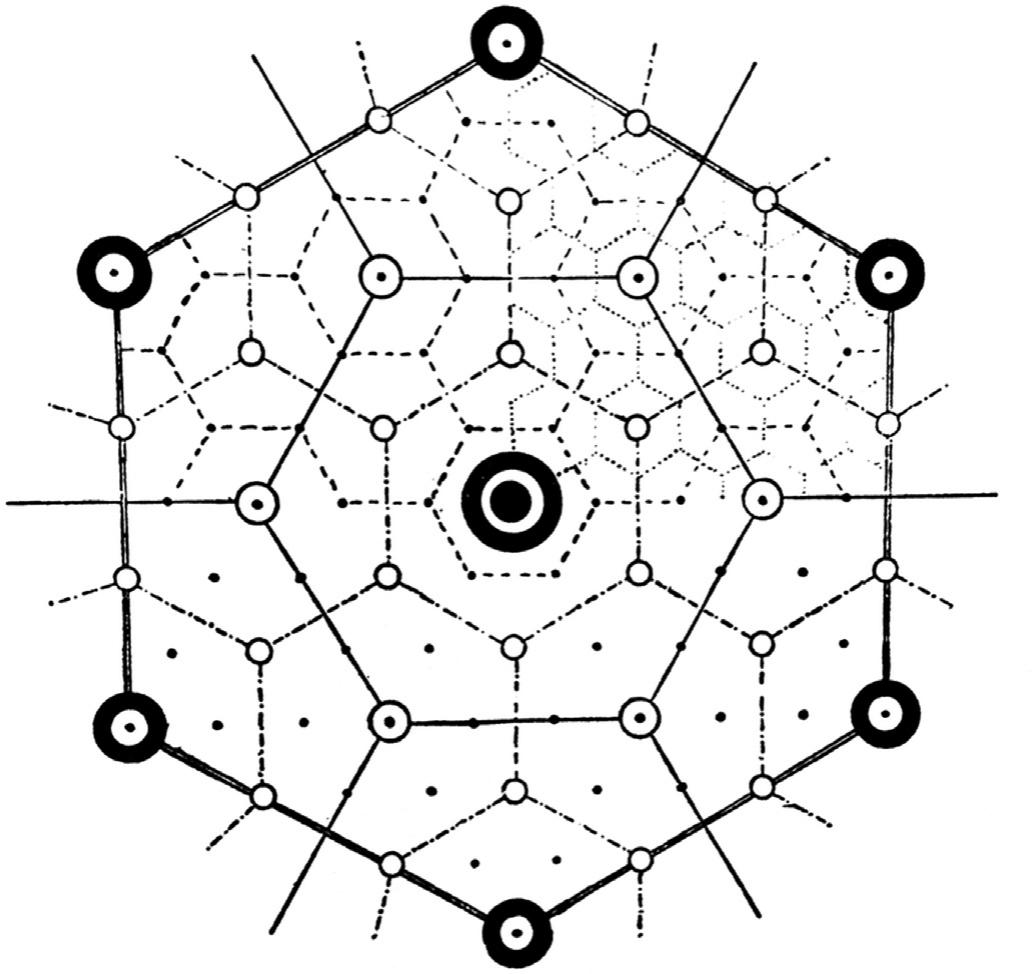
Centres of providing services for different proximal areas - by Christaller 1933.

This project implemented the Central Place Theory which was created by the geographer Walter Christaller, who suggested that swelling units are supposed to work as ‘central places’ to offer services to proximal areas as shown in Figure 6. This theory was discussed in detail by Hildebrand Frey in his book “Designing the City: towards a more sustainable urban form”. A sustainable urban form was suggested along with all its concepts that can work as an alternative for typical city forms towards a more livable sustainable community.

Public transport stops provided accordingly within a walkable distance of homes and workplaces. A modular city structure, a city consists of units that have walkable scale and offer good access to public transport stops. One unit needs to be connected to the other by public transport lines to provide choice of amenities and nobilities. The basis for the micro-structure of the city is the interrelationship of people, transport and amenities. These urban units that offer local facilities within walkable distance are small in scale and population; they only provide day-to-day needs. Any kind of needed service to be provided at a bigger scale can be offered by centres of a higher order of spacial units. These units should be connected by a public transportation system of a higher order. A hierarchical system will be found in the city with regard to the development of clusters (from Fareej to neighborhood) and the transport systems (from bus to Light Railway Train (LRT)). The maximum distance between one dwelling unit and a transport stop is a walkable distance of 10 min. The size of catchment area is an average of 110 ha. The average population density is 60 persons per hectare, which might accommodate around 7,000 persons. Catchment areas for schools, clubs, shops and groceries are not limited to their neighborhood but overlap with other neighborhoods. The concept of building a city from neighborhood units generates a rigid structure that doesn't coincide with the social systems in an open society. This is odd with structure of traditional neighborhood but very close to the very old traditional Arabian neighborhood.

Car use is limited within urban area, the centre of neighborhood could be connected with the centre of Fareej by a bus with stops distributed every 300 m or so. The travel distance from the centre of the district to the edge could be an average of 1,350 m in 5 min. The radius of the central area can have a radius of 150 m.

**Figure 7:**
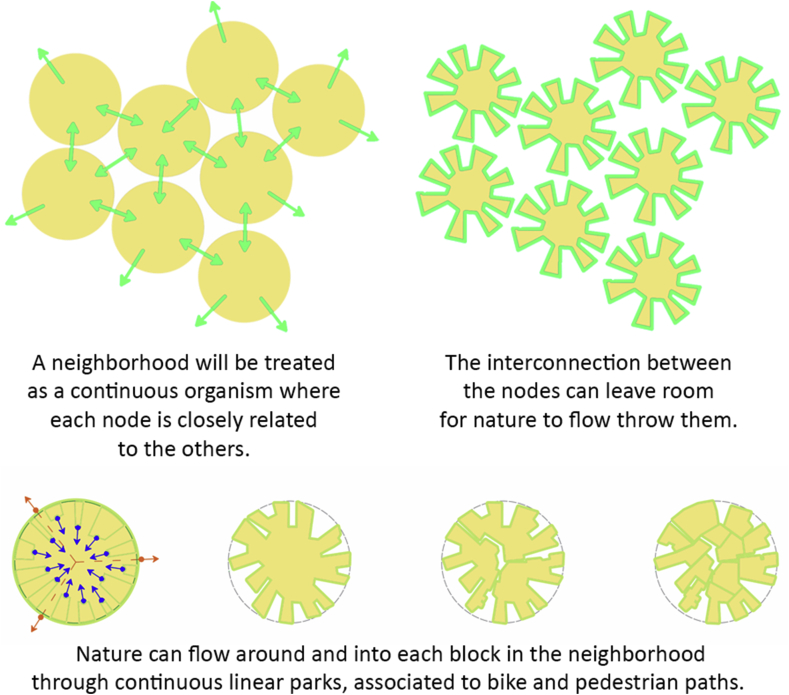
Conceptual diagrams.

There are variety of things to enjoy and activities to serve the city, however, there is either a problem in the connection between that particular activity and our site, or the distance is too long that another similar activity should be provided. On the other hand, some day-to-day needs are better to be provided in each neighborhood with a proximal distance. Such amenities should be provided within a walking distance from user's home. All such day-to-day amenities can be located in one place in the central node of each neighborhood. The conceptual diagrams are shown in Figure 7.

**Figure 8:**
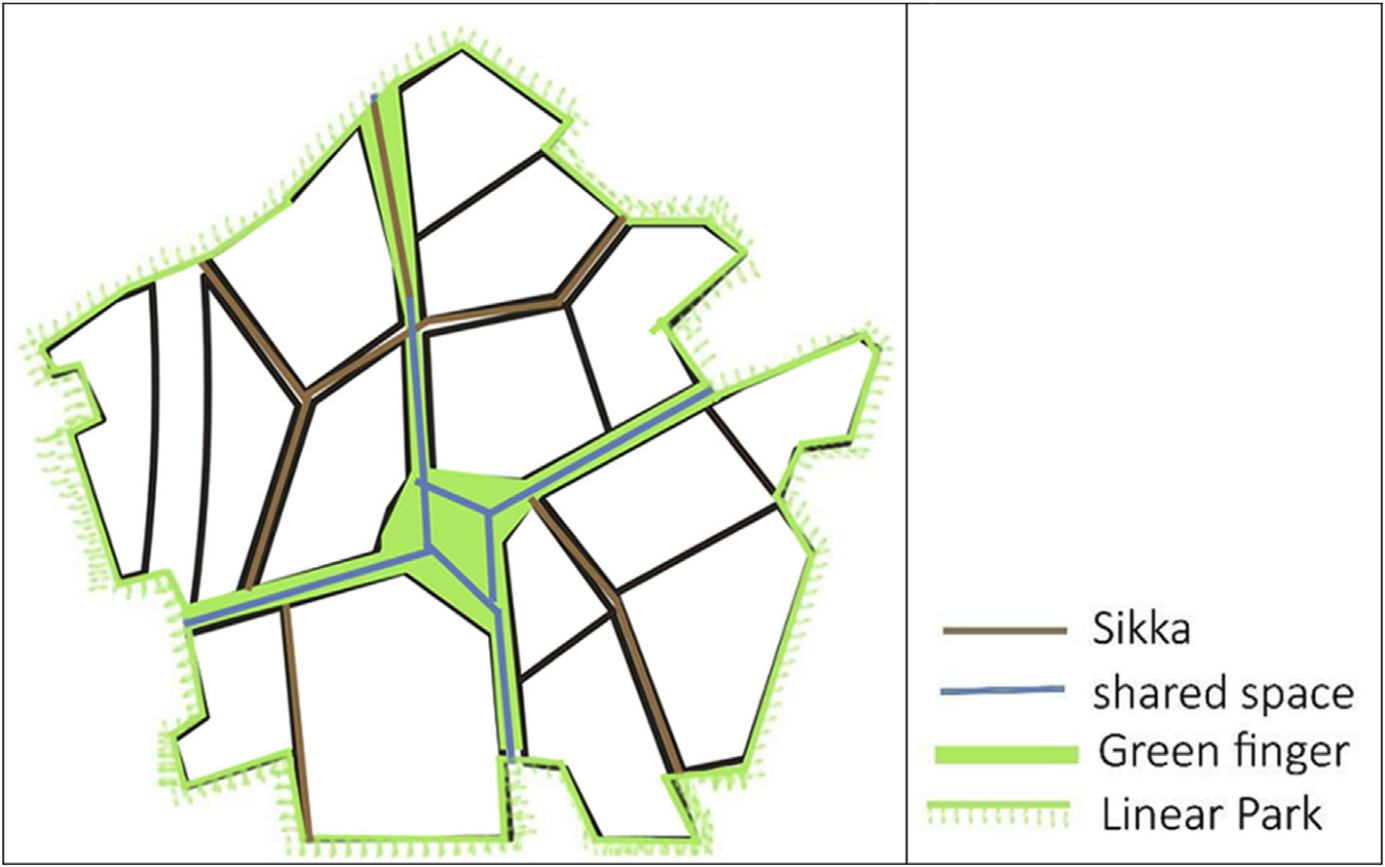
The main Emirati Fareej concepts are applied in the model.

**Figure 9:**
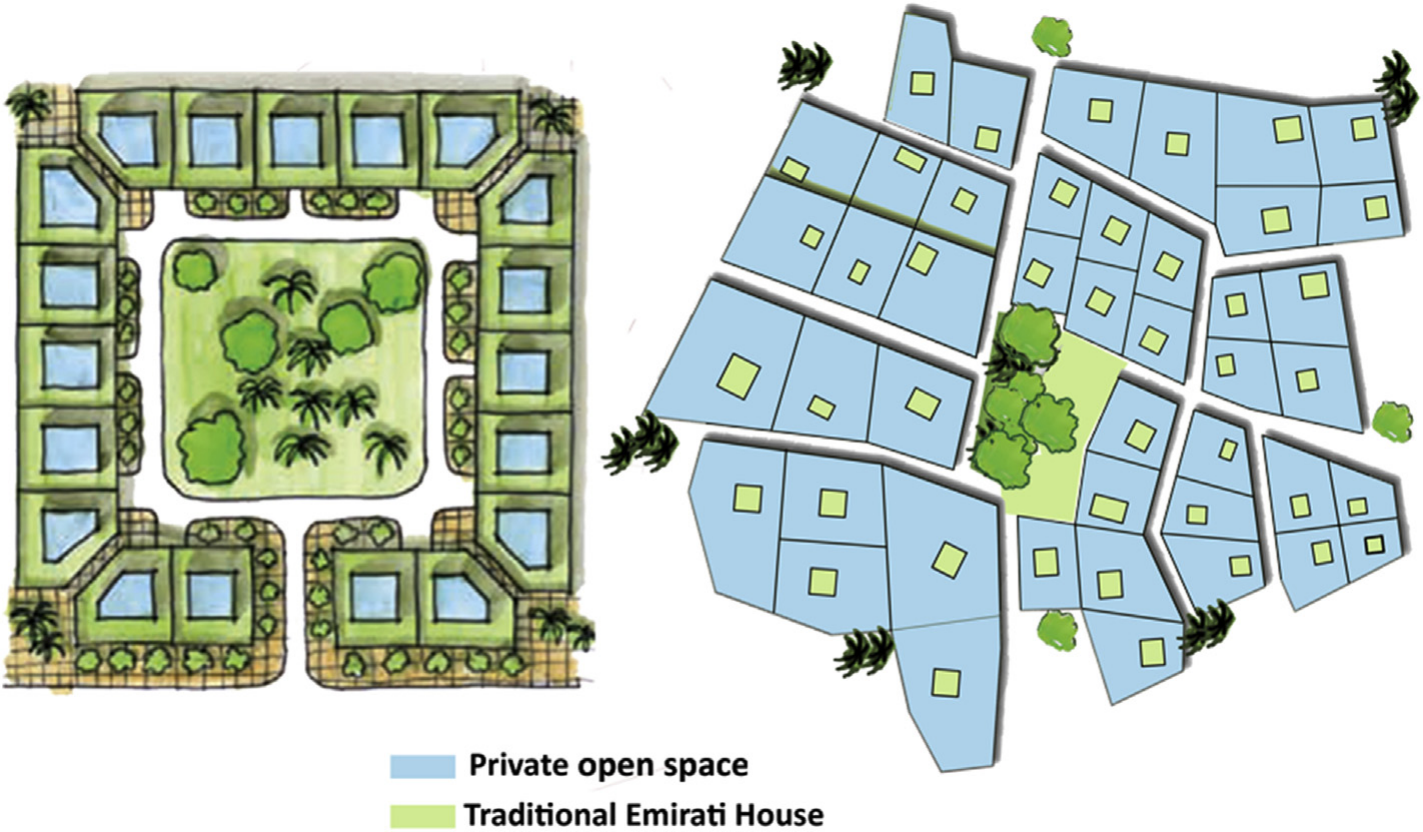
Comparison between the existing Fareej form by UPC and the proposed Fareej form.

Applying such modifications to the normal Fareej structure can lead to a new sustainable form where users are enjoying the green walkways wherever they walk, and these walkways can work as a leading path for them as well. The nodes appear to be in the center of each form. This centre will a pocket park to serve each block it is located in. These nodes shall be connected together through a road/walkway, so the destination will be clear once users leave their home. Moving on to the real model, the form we have is broken down to accommodate the level of interconnection among other Fareej and to provide the maximum green space surrounding the Fareej. This green linear park is located at the perimeter of each Fareej. The Fareej is divided by four main shared spaces. These shared spaces can accommodate green fingers, golf paths, pedestrian walkways and bicycle paths. Another smaller and tighter level of pedestrian occurs at Sikkas. Sikkas are the smallest level of pedestrian walkways that occur among villas and lead the users toward the outer environment of this system. The green fingers aim to connect the green spaces in Al Ain, however, the connection here is done through micro-level as shown in Figure 8. A Comparison between the existing Fareej form by Urban Planning Council (UPC) and the proposed Fareej form is shown in Figure 9.

**Figure 10:**
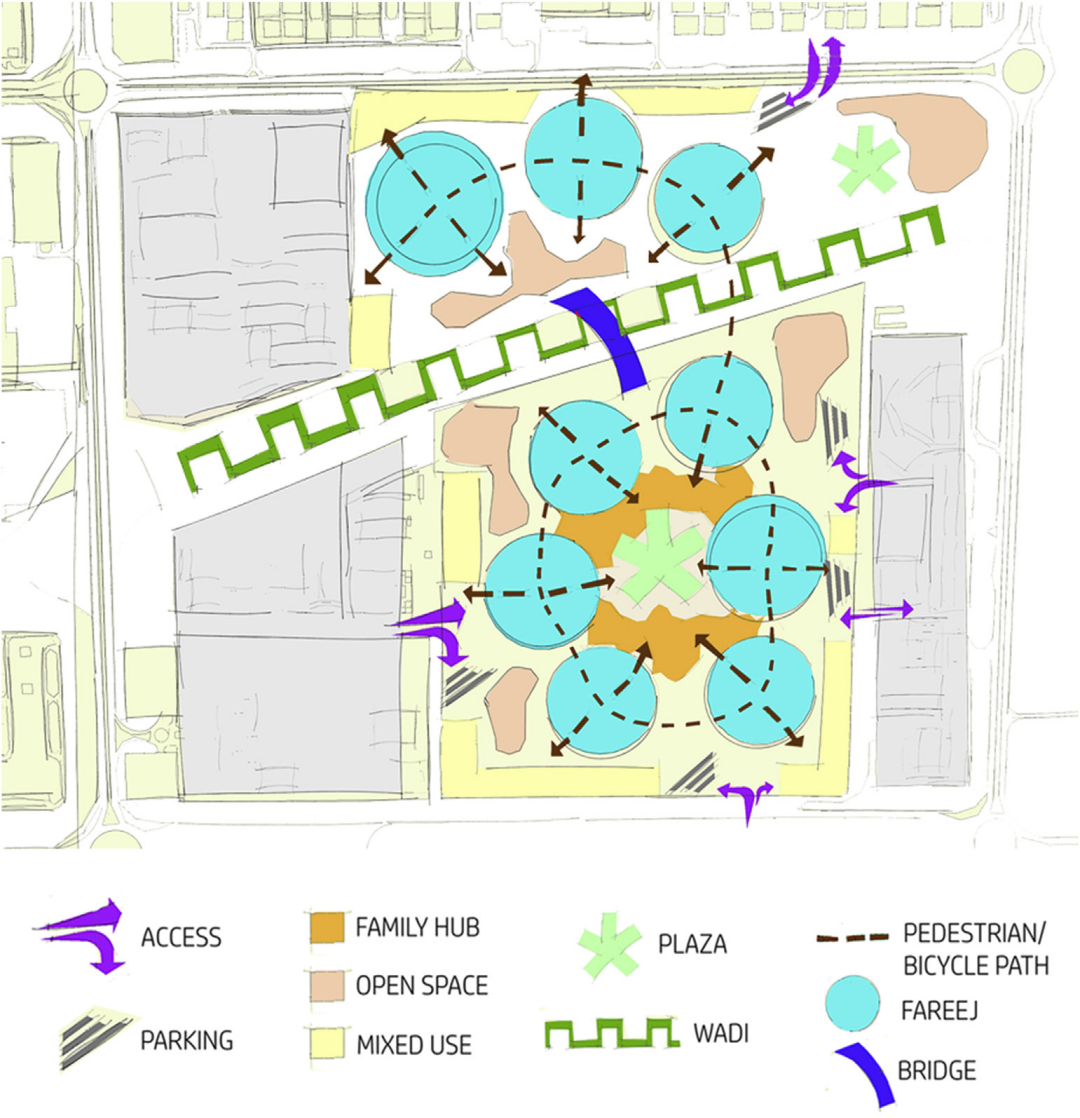
Conceptual plan.

The Family Hub will work as central element that provides the daily needs of users within the neighborhood catchment area. The interconnectivity between the multiple Fareej creates an ecosystem neighborhood that interacts strongly with its surrounding as well as its core, the Family Hub as shown in Figure 10.

## 4 Results and discussion

The rationale in having another alternative in developing the urban space other than the one suggested by the Urban Planning Council, is to recreate urban connections while preserving the historical features of a community at the micro-level. The need to preserve the authenticity of Al Ain city through having a Fareej community was successfully achieved at multiple levels. Firstly, the provided alternative layout for each unit is to have the Emirati dwelling style with private courtyard. This should have a better impact than the layout of the open yard suggested by the Council. Secondly, at the city planning level, Al Ain city has natural resources presented mainly in the oases. These oases were successfully connected with our suggested plan to ensure the continuity and connectivity of the designed community within the city. The green fingers that occur at one landscape layer within the Fareej community ensure a very important connectivity feature that shouldn't be overlooked when revitalizing a space within a city. This was clearly reflected by the integration of landscape at the micro-level of a dwelling unit towards the macro level of a city.

The existing urban form of the site under study is scattered and dispersed. Some development occurs on the site, however, this kind of development of undefined form. There is an obvious waste of space and sense of lost. This results in unpleasing space to live or work in. The superblocks that exist in the site should be broken down into smaller blocks each of an average length of 120 m before a user can turn around. Those blocks should define both sides of the road. Roads should be defined by the blocks while respecting the surrounding development. Public open spaces should have a unified form as well, and should occur in consistent with the surrounding blocks. Whenever there is an existing open space, an open space should be developed in the site with respect to the existing one and adjacent to it.

In the proposed urban form, the plan looks much densified with uniformed blocks. The development occurs to look more compact in comparison with the existing. The high-density residential villas appear to have a very unique form with open spaces that are well connected. The form that is applied in the areas of Fareej are following the central space form. The form allows the designed to place a node in the centre of each form. This node is indicated with white color in the map to represent the role of it with is an open space for people of the Fareej to gather. It is obvious from the proposed plan that this node is connected to each of the blocks of the same Fareej and to the surrounding blocks. One of the important spaces is the one that occurs in each Fareej towards the central area that is called in this project “The Family Hub”.

**Figure 11:**
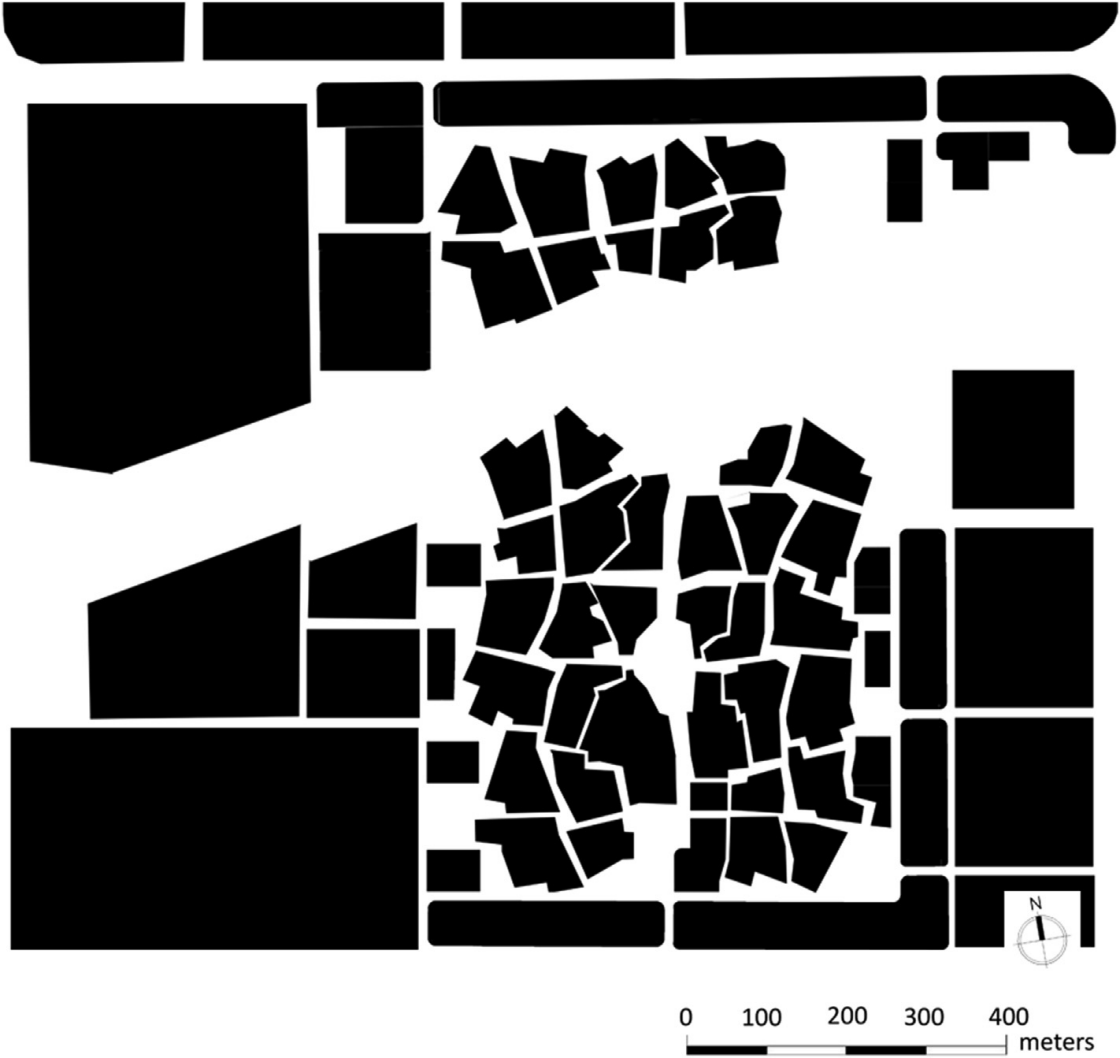
Block structure.

The typologies of the blocks that occur on the perimeter of the site are very systematic and well responding to the surrounding. These blocks are of a different use, they are mixed use G+4 developments as shown in Figure 11.

**Figure 12:**
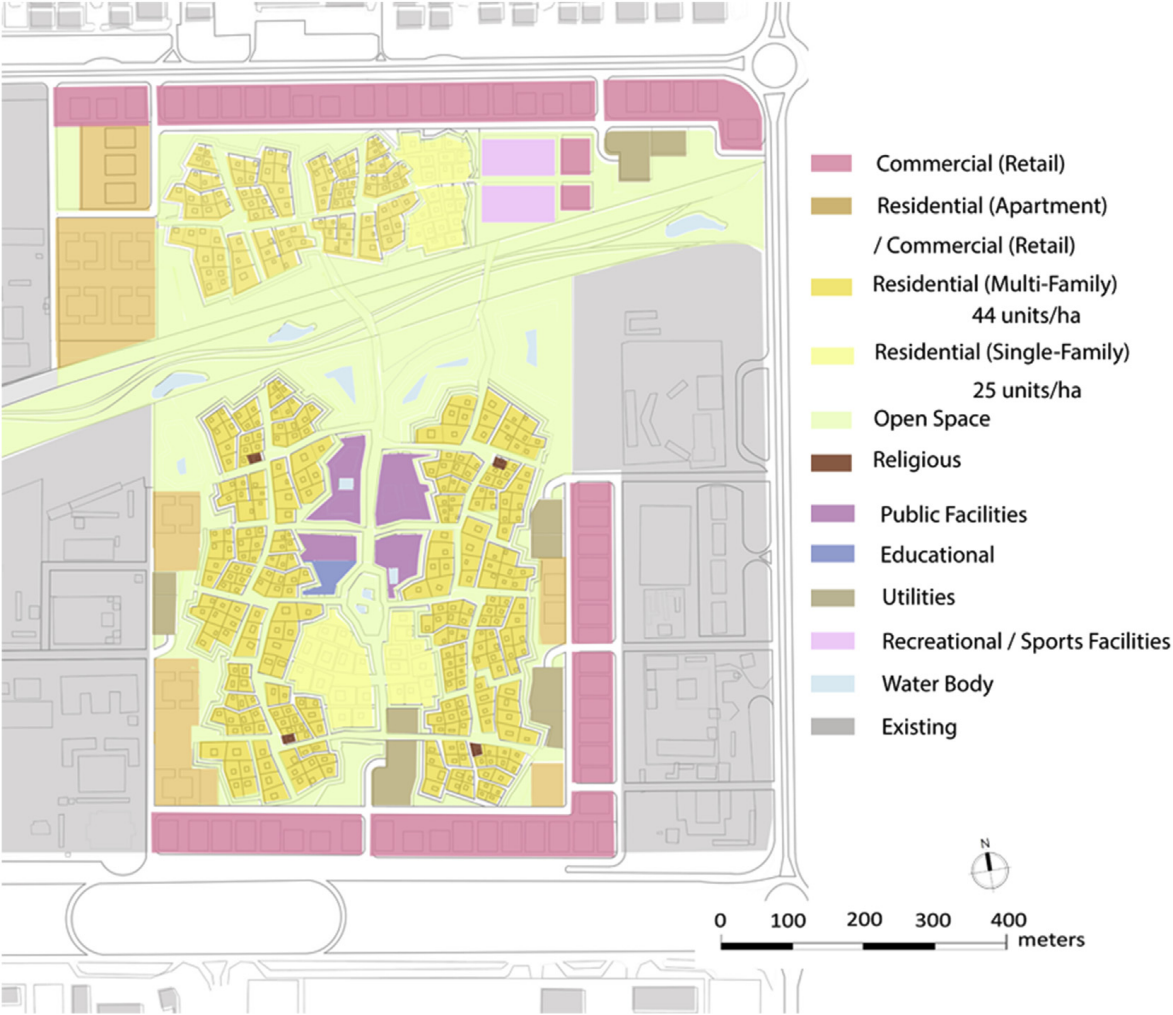
Proposed land use plan.

Analysing the surrounding activities and existing amenities and recreational facilities, leads us to plan the requirements for the site and to plan for their accessibility to the city. Some daily needs can be provided in a proximal distance within each neighborhood and within a walkable distance from a user's dwelling unit. As shown in Figure 12, the land use distribution within the site follows the Central Place Theory.

**Figure 13:**
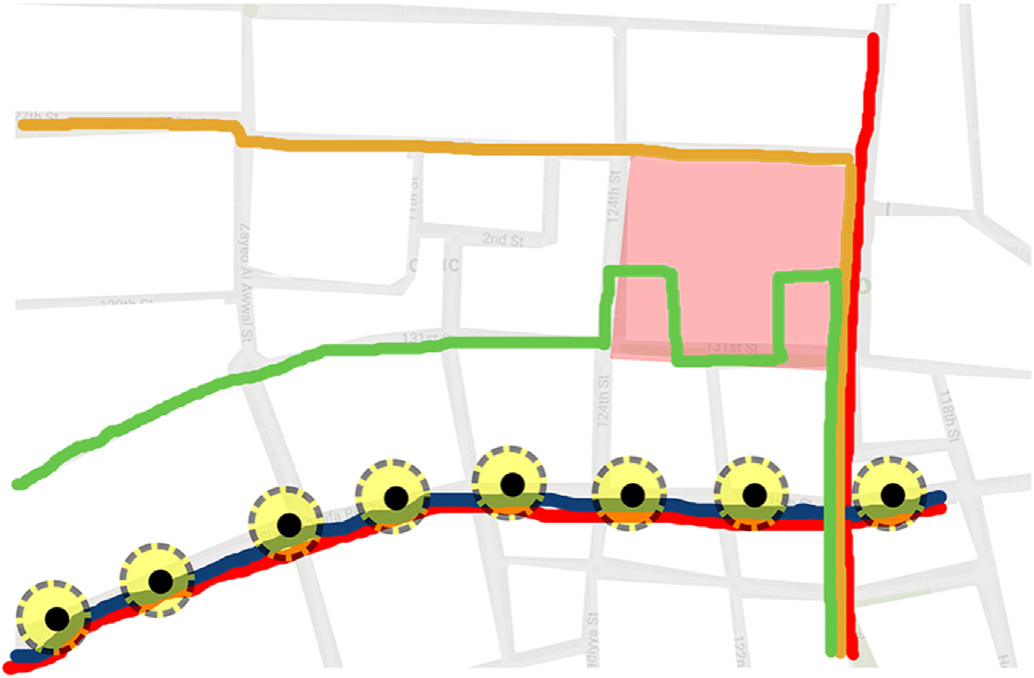
Diagrammatic connections with Al Ain 2030.

**Figure 14:**
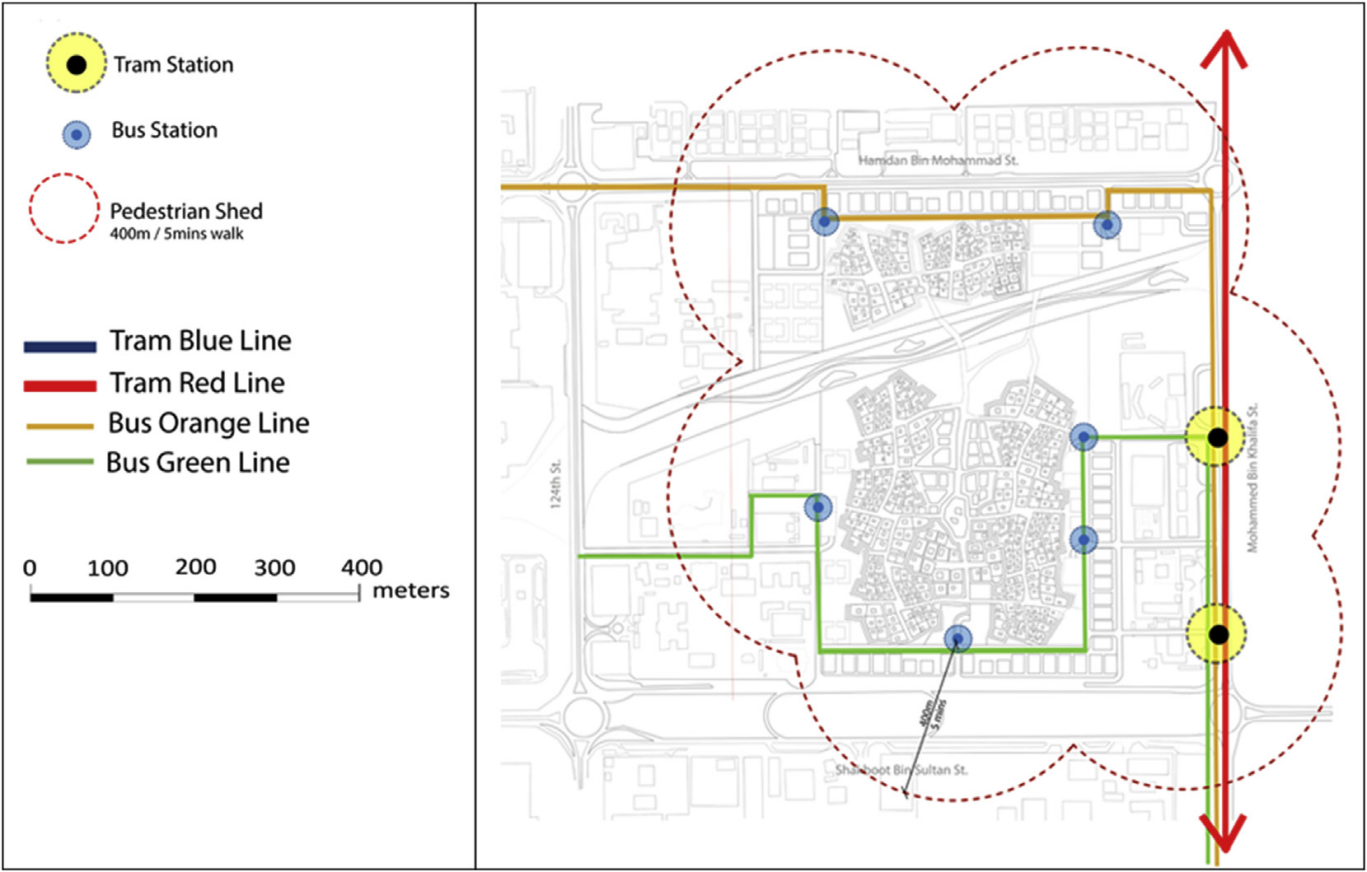
Vehicular street & non-motorised network.

**Figure 15:**
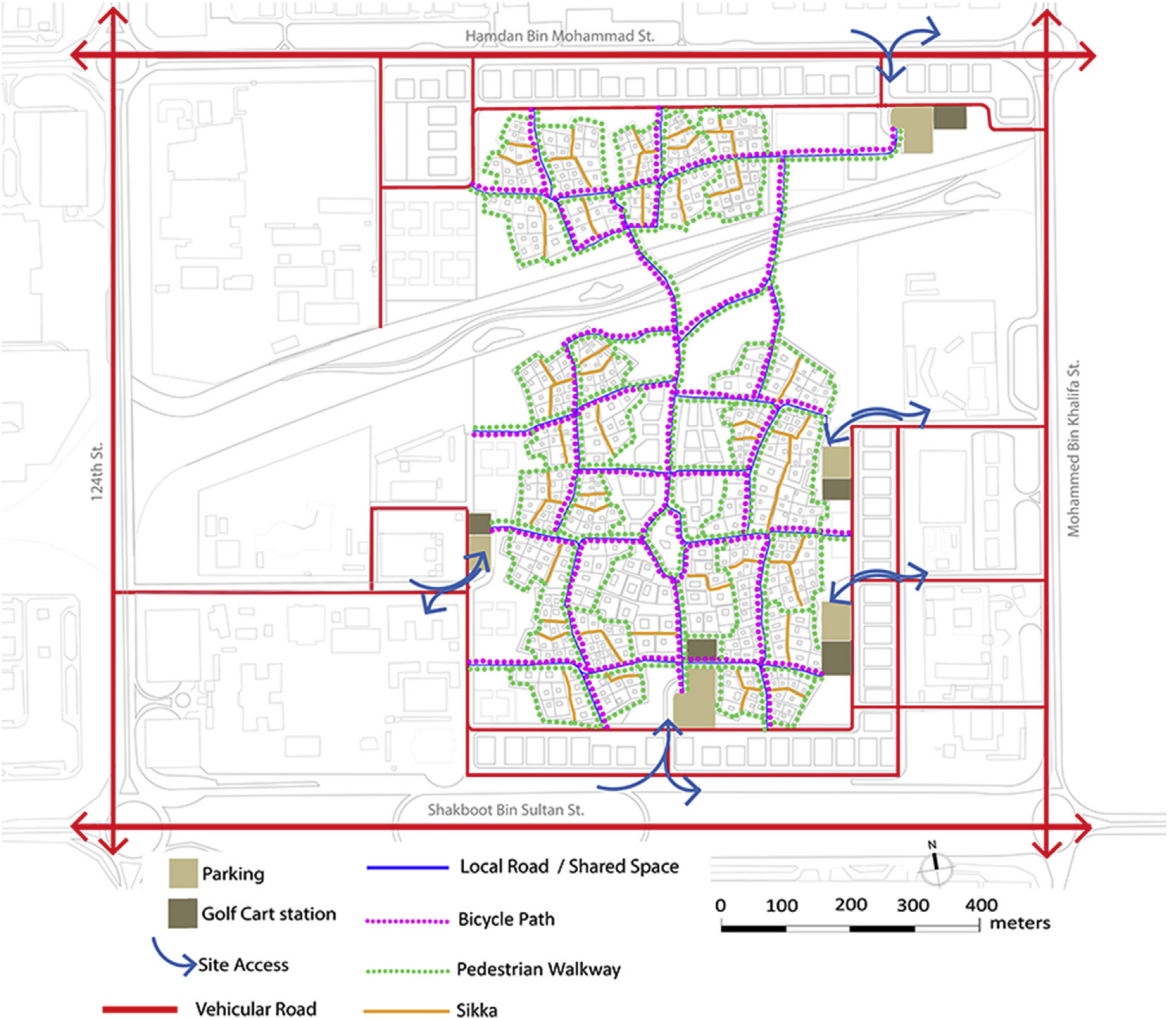
Vehicular street & non-motorised network.

The Fareej Community proposed in this project is a zero-car community. Roads within the site under study are all considered to work as shared spaces. This means they should accommodate pedestrian, cyclists, golf carts, etc. Users should be able to get to any destination within the site easily by foot, bicycle or a golf cart. There is another level of pedestrian walkways that occur between the residential units, it is called Sikka. Sikkas are a traditional Emirati element in the Fareej form that occurred in the historical spaces in the city. On the other hand, there are public transit connections that serve the site as well. A proposed tram by plan Al Ain 2030 is connected to the site under study through two bus lines as presented in Figures 13, 14, and 15.

**Figure 16:**
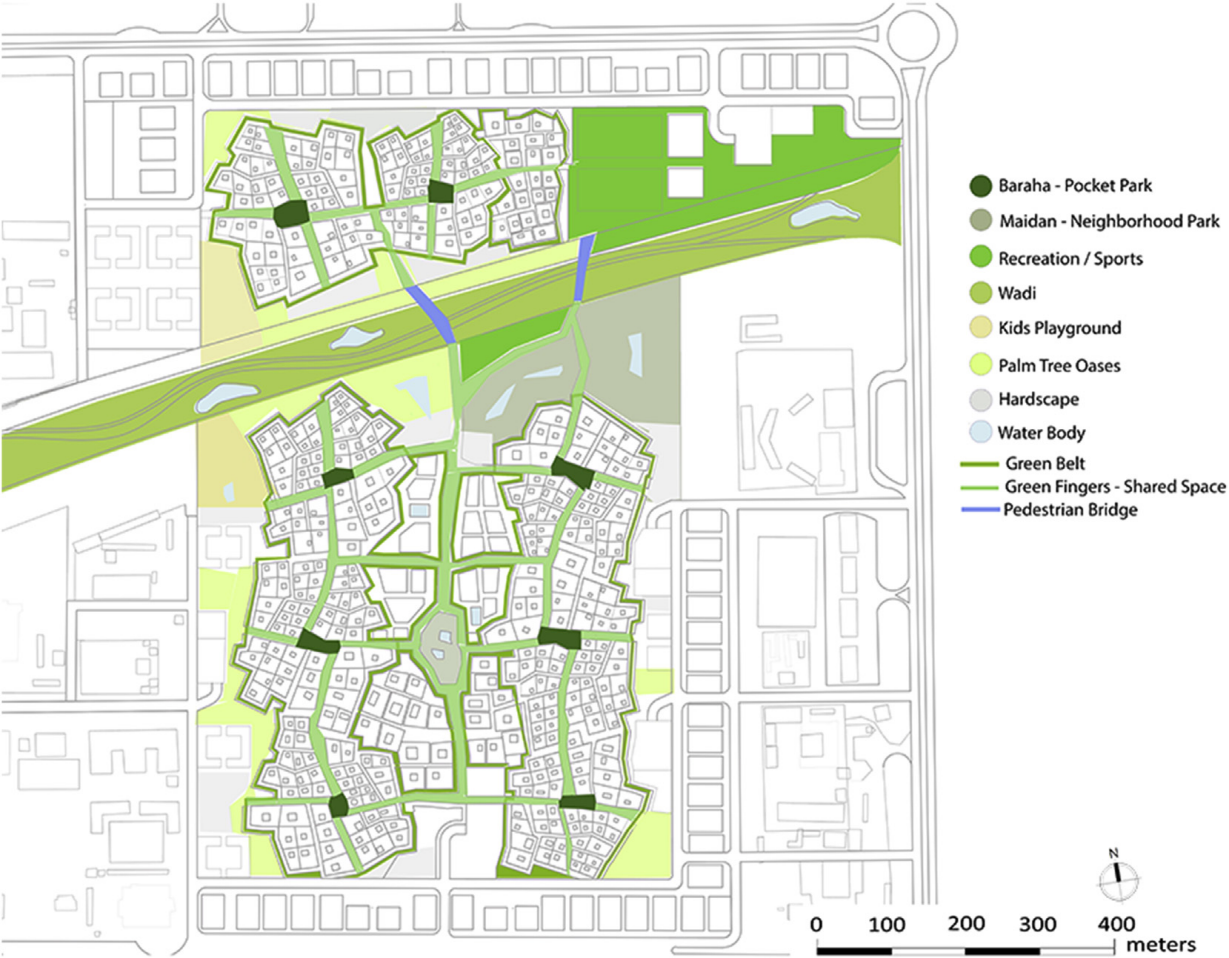
Proposed open space plan.

One of the major issues found in the site under study was the vacant lands with undefined function. Another issue was found in Al Ain city, in general, was the disconnected open spaces. These two factors were acting behind the concept of open spaces in this project. Suggesting well treated open spaces that are well connected together is one solution. Some of these open spaces will work as green fingers. This concept was suggested by the UPC. Green fingers are green pedestrian sideways to connect the oases within the city. The type of connection that should be happening here can be applied in a micro level. Privacy for the neighborhood will define another level of green connections. These green fingers can work for another function within the neighborhood. They can connect the different pocket parks (barahas) in each Fareej. They can also work as a connection between a bara-ha and a maidan (plaza). A green edge surrounding each Fareej can work as further recreational facility for people to enjoy while heading to their destinations. Engaging such level of green space in the site can enhance the quality of life within the neighborhood. Other types of open spaces occur in the proposed open space plan as shown in Figure 16. These open spaces varies between public and semi-public. The smallest gathering space available in the site is the pocket park. The plaza is bigger in size and can serve the whole area of neighborhood. A sports open space that is located on the north of the wadi can serve the whole neighborhood as well. This can function as a main space for users to enjoy playing football, basketball, and other outdoor sports. A jogging track and a bicycle lane of unobstructed linear path is available in the new plan of revitalizing the wadi. A plaza that is located in the centre of the neighborhood within the family hub can work as a very important gathering centre. A well-treated open space is the key to plan for better communities.

**Figure 17:**
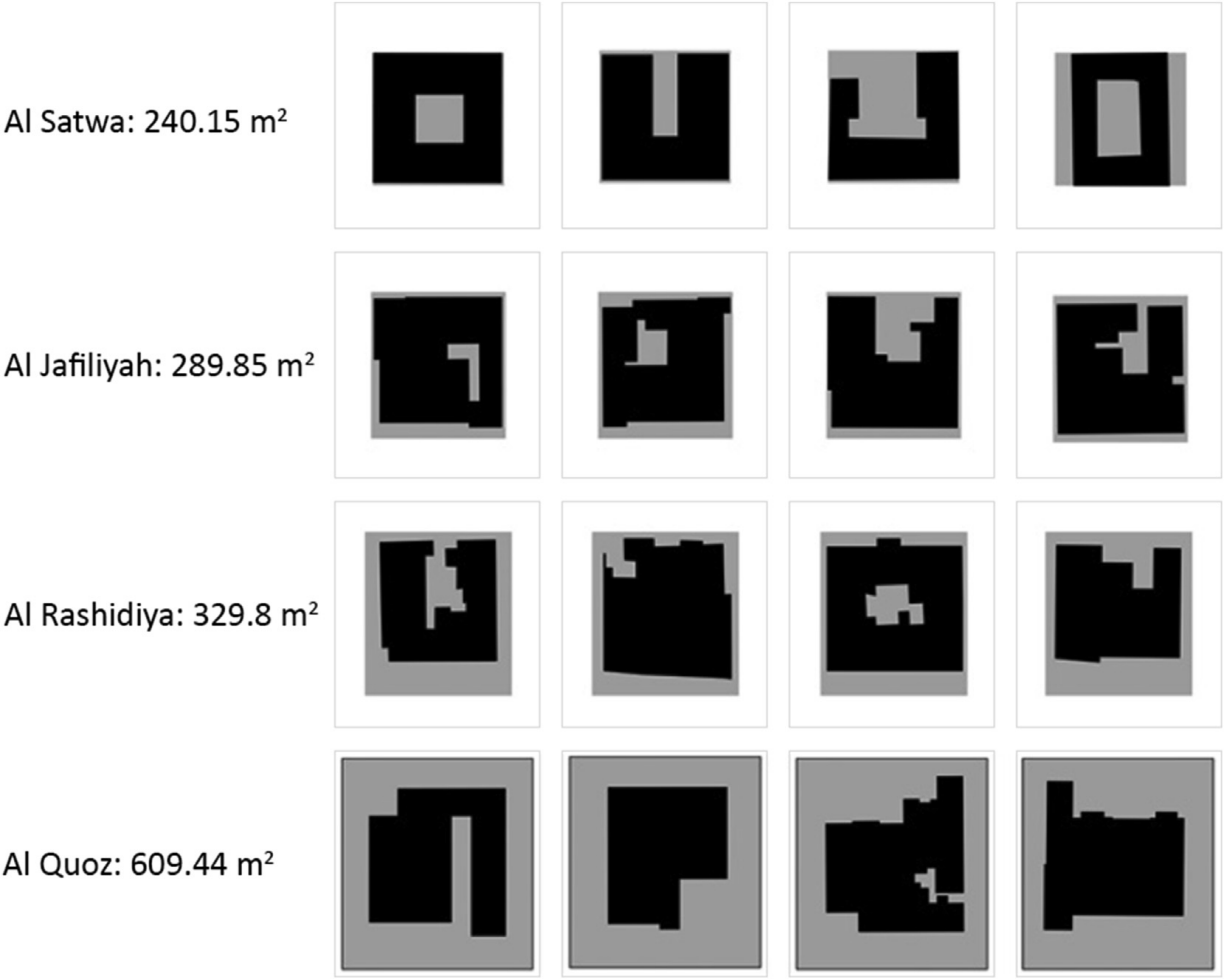
A traditional dwelling unit evolution in morphology. The transformation happens also at size and area. The full utilization of land is seen in the first three rows, while the bottom row shows houses surrounded by a fence. This transformation took place in the 1970s (Asim Khanal and Khaled Alawadi) [28].

**Figure 18:**
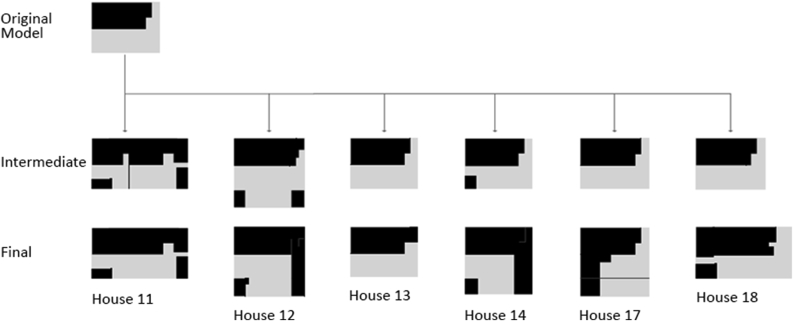
Morphological transformation of dwelling unit of a certain type in Al Hili district of Al Ain (based on drawings from Al Ain Municipality, Engineering Section and Al Dhaheri, 1999) [28].

**Figure 19:**
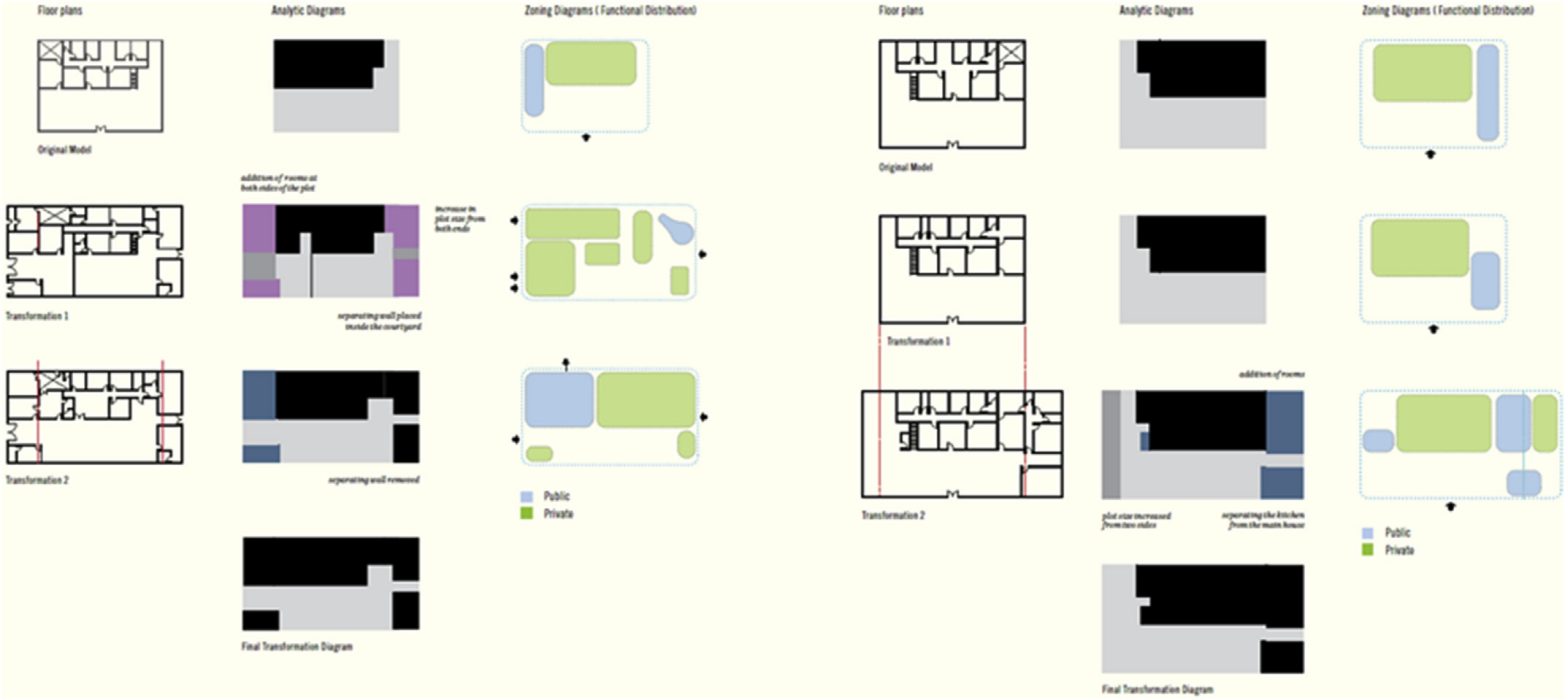
A detailed analysis for two types of dwelling units [28].

**Figure 20:**
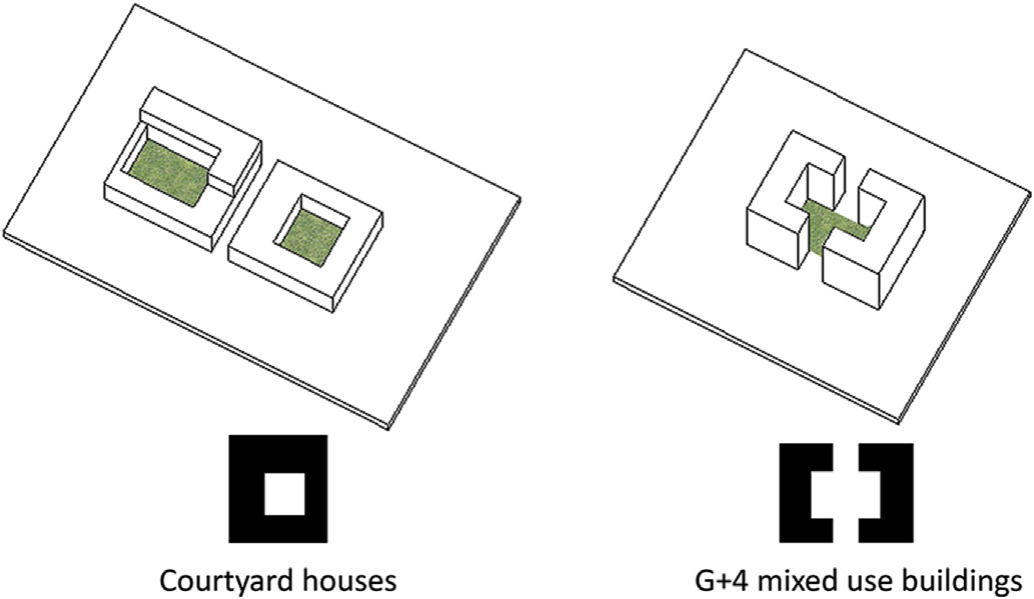
The different typologies of the dwelling units used in the project under study.

Major differences are valid to distinguish between the traditional and contemporary urban fabric in the UAE as shown in Figures 17, 18, 19, and 20. The differences occur at multiple levels; form, space, and social needs. The traditional communities were of high density, walkable, well connected, easy oriented, social inviting, and mixed-use services. It also enhanced the need of privacy for users of each dwelling, the need of housing extended family members comfortably, and fulfilling the social needs of the citizens through providing communal spaces. It is known that traditional communities are a functional livable system at various levels. Another feature is the connection of roads and streets at multiple levels (alleys to main roads) and these weaving paths help in locating land uses to occur at proximal distances within one community (Fareej).

Major changes in the system occurred during the 1970s, these changes were linked to the intentions of better provision and a luxury of living. The natural decentralized system was replaced by fewer connected road network and elongated blocks. The appearance of private surrounding fences and gates took place as well and it added on the disturbance of the traditional urban form. This transformation was a result of changing policies of zoning and regulations in order to accommodate larger plots and spaces for the growing number of citizens. One main influence was the governmental settlements to accommodate citizens. In this scenario, the architects working on such projects used to be expats and their knowledge about the traditional framework was limited. This resulted into losing the traditional aspects of architecture in the country beside the frequent change of the dense urban fabric. The number of housing projects that were spread across the country looked the same, regardless of the location or climate. For example, the courtyard that was strongly emphasized in the traditional architectural dwelling unit, it was partially lost later in the contemporary approach. This was not only a discarded architectural feature but also a loss of a family communal space. This courtyard appeared in later contemporary prototype as a smaller space, where the area is added for extra room in the enclosed space. Another lost feature was the alignment to alleys through interconnected grids. The dwellings followed the geometry available on site and were always connected to one level of the road network. In the contemporary urbanized system, the city starts with a systematic grid and dwelling units follow this grid regardless whether or not it is directional and serves the proximity measures of the provided services within a walking distance. The contemporary approach into designing dwellings set them up equal in size, height, courtyard location and entrance direction. This monotonous approach left the dwellings without any identity not even to relate to the social hierarchy. This's another lost feature that made the cities car dependent. The alleys were lost and the single community (Fareej) was less connected making it hard to accommodate daily needs without the use of a car.

**Figure 21:**
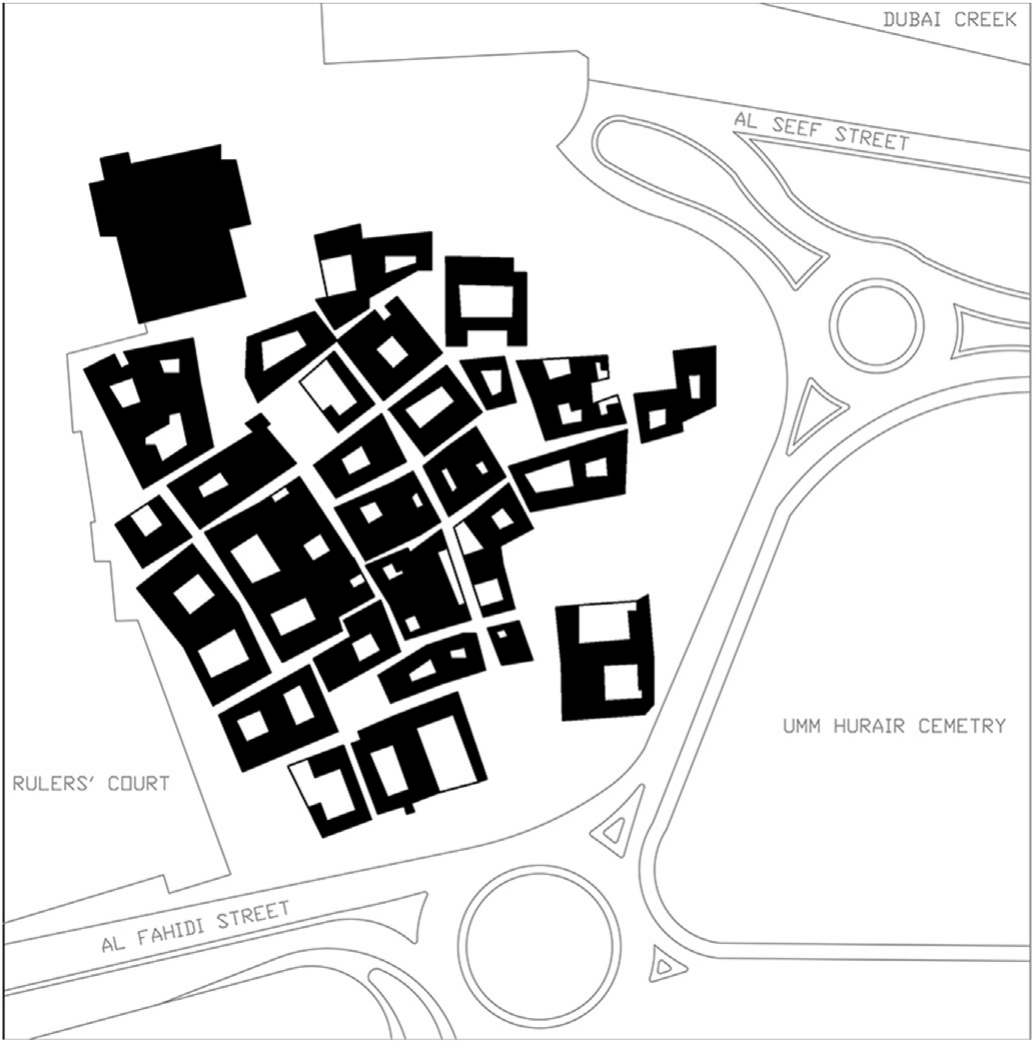
Al Bastakiya figure-ground.

Describing the urban fabric formed from a traditional dwelling unit, Al Bastakiyyah in Dubai (shown in Figure 21) is a good example for highlighting this. It is one of the last built historical districts in the UAE with all the traditional aspects. The construction of the district took place in 1890s. It models a traditional Fareej community with courtyard units. These units built to the edge of parcel for maximum occupancy. The alleys existed to connect the units to each other and to other facilities. This district accommodates multiple functions and is normally busy with users enjoying their walk around the site. The site was reconstructed according to its historical value. Nowadays, this district plays an important role as an authentic still-living district.

The same approach applies to Al Ain Civic Centre. The invitation of getting back in time to use the traditional urban elements is a new request by decision-makers in the UAE; Abu Dhabi Urban Planning Council. It is a call from the government to focus on using such concepts into working on major projects to better reflect the image of the city. The preservation of any historical elements in the site is a priority before infilling the land with the discussed kind of fabric. The infill work discussed in this paper happens at various levels; the historical/traditional identity of the city, the urban fabric of the city and the typology of a single dwelling unit. The provided design proposal was not only dependent on a nostalgic historical design but also on emphasizing the need to revive the local architectural vocabularies. Al Ain is a resilient city and this should be certainly reflected on its image. A community urban fabric is established to be resilient by testing it through climate change and context. The Emirati society deserves to re-live the quality urban spaces that once existed in their ancestors' lives. The tradition of this society plays a great role in the lives of its citizens. A deep kind of connection between the old and present should be visible in the image of the city as well as the daily life of the users.

## 5 Conclusions

**Figure 22:**
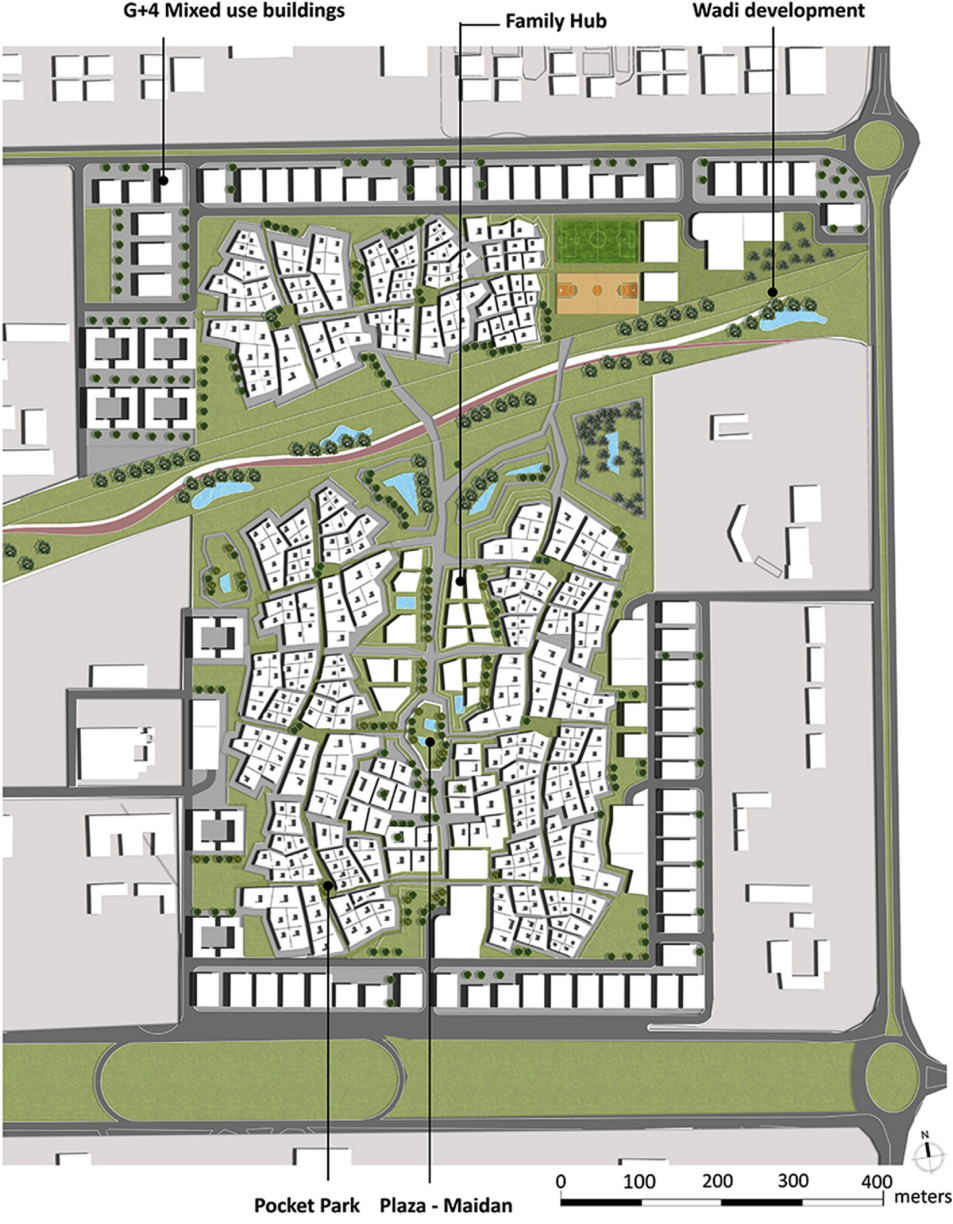
Master plan.

**Figure 23:**
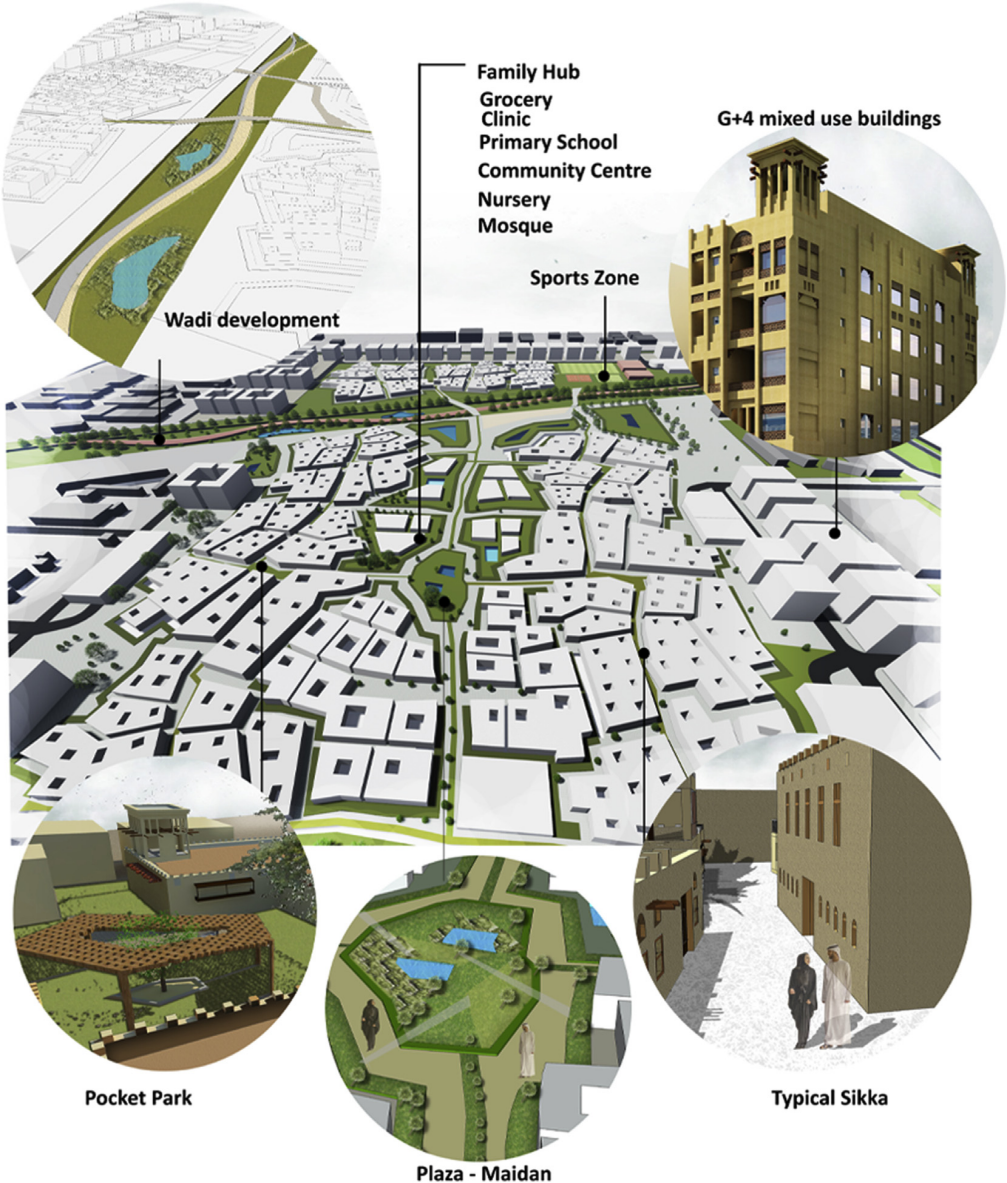
Birdseye view.

The proposed master plan of the project represents a master-planned Fareej Community. The residential neighborhood with its traditional Emirati Fareej form is spread among the site. The Fareej form is adjusted to serve the lifestyle of users nowadays. Having a central space is certainly serving the whole neighborhood with proximity to all the facilities. The recreational and functional open spaces are available everywhere around the site. Bike paths and pedestrian walkways are also connected all over the site. All roads work as shared spaces with access limited for golf carts only. There will be a distinguished difference in the appearance and function of the previously undeveloped area and the proposed master plan as presented in Figures 22 and 23.

## Declarations

### Author contribution statement

Inshirah Shublaq: Conceived and designed the experiments; Performed the experiments; Analyzed and interpreted the data; Contributed reagents, materials, analysis tools or data.

Rafael Pizarro: Conceived and designed the experiments; Contributed reagents, materials, analysis tools or data.

Abeer Abu Raed: Analyzed and interpreted the data; Contributed reagents, materials, analysis tools or data; Wrote the paper.

Tamarah Alqalami and Esraa Altwassi: Contributed reagents, materials, analysis tools or data.

### Funding statement

This research did not receive any specific grant from funding agencies in the public, commercial, or not-for-profit sectors.

### Data availability statement

No data was used for the research described in the article.

### Declaration of interests statement

The authors declare no conflict of interest.

### Additional information

No additional information is available for this paper.

## References

[1] UNESCO (1976).

[2] ICOMOS (1987).

[3] Torre M.d.l., Avrami E. (2000).

[4] Orbasli A. (2020). Urban Conservation.

[5] Van Dun P. (1993). ICOMOS International Scientific Committee.

[6] Van Dun P. (2001). http://www.arcchip.cz/w03/w03_dun.pdf.

[7] Tiesdell S., Oc T., Heath T. (1996).

[8] UNESCO (2011). Paper Presented at the UNESCO General Conference at its 36th Session Paris.

[9] Barthel-Bouchier D. (2013).

[10] Zeayter H., Mansour A.M.H. (2018). Heritage conservation ideologies analysis – historic urban Landscape approach for a Mediterranean historic city case study. HBRC J..

[11] Doratli N. (2005). Revitalizing historic urban quarters: a model for determining the most relevant strategic approach. Eur. Plann. Stud..

[12] Muminović E., Radosavljević U., Beganović D. (2020). Strategic planning and management model for the regeneration of historic urban landscapes: the case of historic center of Novi Pazar in Serbia. Sustainability.

[13] Asfour Khaled (2007). Polemics in Arab architecture: theory vs. practice. Archnet–IJAR Int. J. Architec. Res..

[14] Edwards Brian, Sibley Magda, Hakmi Mohammad, Land Peter (2006). Courtyard Housing: Past, Present, and Future.

[15] Glendinning M. (2013).

[16] Hakim Besim S. (2007). Revitalizing traditional towns and heritage districts. Archnet-IJAR: Int. J. Architect. Res..

[17] The European Parliament: Historical Background (1975). http://www.europarl.europa.eu/ftu/pdf/en/FTU_1.3.1.pdf.

[18] Van Oers R., Pereira Roders A. (2012). Historic cities as model of sustainability. J. Cult. Herit. Manag. Sustain Dev..

[19] Bandarin F., Van Oers R. (2012).

[20] Getty Conservation Institute (2010). http://www.getty.edu/conservation/publications_resources/pdf_publications/pdf/experts_mtg_mar09.pdf.

[21] Tweed C., Sutherland M. (2007). Built cultural heritage and sustainable urban development. Landsc. Urban Plann..

[22] Evans G. (2005). Measure for measure: evaluating the evidence of culture's contribution to regeneration. Urban Stud..

[23] Jacobs J. (2011).

[24] Lynch K. (1960).

[25] Abu Raed Abeer (2018). Rehabilitation of industrial sites: economical and social aspects. J. Eng. Appl. Sci..

[26] Carta M. (1999).

[27] UN-HABITAT (2011).

[28] Ali Esraa, Al Dakheel Joud, Ghatrif Lama Al, Rahmani Meriem (2016).

